# Editorial: Community series in the role of CD1- and MR1-restricted T cells in immunity and disease: volume III

**DOI:** 10.3389/fimmu.2026.1783936

**Published:** 2026-01-26

**Authors:** Kazuya Iwabuchi, Luc Van Kaer

**Affiliations:** 1 Department of Immunology, Kitasato University School of Medicine, Sagamihara, Japan; 2 Department of Pathology, Microbiology and Immunology, Vanderbilt University School of Medicine, Nashville, TN, United States

**Keywords:** CD1, immune-mediated disease, immunity, MR1, mucosal-associated invariant T cells, natural killer T cells, non-peptide antigens, unconventional T cells

Cluster of differentiation 1 (CD1) and major histocompatibility antigen (MHC) complex-related protein 1 (MR1) are members of the MHC class Ib family of antigen-presenting molecules that control the development and functions of unique subsets of T lymphocytes (1–6). CD1d and MR1 expression by CD4^+^8^+^ thymocytes facilitates the development of invariant natural killer T (iNKT) cells and mucosal-associated invariant T (MAIT) cells, respectively. These T cell subsets display a highly restricted T cell receptor (TCR) repertoire, with hardwired effector functions reminiscent of cells of the innate immune system, a property they share with γδ T cells (2, 7). These three unconventional T cell subsets may influence each other by competing for homeostatic factors or niches, and potentially display redundant functions (8). The unique properties of these innate-like T cell subsets were discussed in Volumes I and II of this Community Series. Volume III, which contains one original research article and five review articles, provides examples of the most recent work in this field.

The review by Rotolo et al. discusses the therapeutic potential of iNKT cells in cancer, infections, and graft-versus-host disease (GVHD). The anti-tumor activities of iNKT cells and their prototypical ligand, α-galactosylceramide (α-GC), have been well documented (9, 10). The review article discusses encouraging results of clinical trials with iNKT cells. The authors discuss the feasibility of employing allogeneic, off-the-shelf iNKT cell preparations, since these cells do not mediate GVHD and might even suppress alloreactivity. Recent progress in this field includes clinical preparations of various engineered iNKT effectors such as chimeric antigen receptor (CAR)-modified iNKT cells, induced pluripotent stem cell (iPSC)-derived iNKT cells, and iPSC-CAR-iNKT cells, which may accelerate the development of safe and effective iNKT cell therapies. Moreover, the review discusses iNKT cell-based combination therapies with radiotherapy, monoclonal antibodies, immune modulators, and others.

The review by Laub et al. covers tumor immunotherapy by mainly focusing on MR1-restricted T cells, as compared with iNKT cells and γδ T cells. The advantage of utilizing unconventional T cells for immunotherapy is based on their public TCRs that recognize non-peptidic antigens presented by non-polymorphic or minimally polymorphic MHC class I-like molecules. While MR1 is ubiquitously expressed, it is largely confined to intracellular compartments in the absence of specific ligands (11). Hence, target cells for MR1-restricted T cells need to produce endogenous activating ligands. MR1-restricted T cells can be activated or inhibited by a variety of self-, tumor-, or microbial-derived products and drug-like molecules such as vitamin B-related metabolites, nucleosides, nucleoside adducts, and their synthetic analogues. The authors discuss the properties of human MR1-restricted T cell clones that recognize specific MR1 allomorphs on a wide range of tumor cells but not healthy cells, suggesting a new avenue for MR1-based immunotherapies. Finally, they discuss the current status and future perspectives of clinical trials for cancer immunotherapies conducted with these unconventional T cell subsets.

The review by Palacios et al. discusses the function of iNKT cells in humoral immunity via direct and indirect interactions with B cells, as facilitated by iNKT cell ligands. They review various α-GC analogues that preferentially activate type 1 or 2 immunity, anatomical structures of the secondary lymphoid organs that facilitate iNKT-B cell interactions, intricate cognate and non-cognate interactions between these cells, and their roles in pathological settings such as autoimmunity, infection and metabolic disease. They propose that interactions between iNKT cells and B cells mediated by specific ligands could be exploited to induce specific immunoglobulin isotypes and effector functions to advance vaccine development.

Seminal studies in the field reported the capacity of members of the CD1 family to present mycobacterial lipids to CD1-restricted T lymphocytes (12). These studies spurred the discovery of a burgeoning family of unconventional T cells that recognize non-peptidic antigens presented by MHC class Ib proteins. The review by Milton and Mansour describes the antigen-presenting functions of each member of the CD1 family, and further discusses the responses of subsets of CD1-restricted αβ and γδ T cells to mycobacterial infections and autoantigens. They also highlight earlier findings that different mammalian species express different numbers of CD1 genes, which has important implications for selecting appropriate animal species to model disease (13). Importantly, they remind readers that toll-like receptor signaling can modulate CD1-mediated T cell responses, including those directed against mycobacterial infections. Prospects for incorporating this knowledge into new vaccine candidates are discussed.

MAIT cells can be activated by cytokines such as IL-12 and IL-18, even in the absence of TCR signals (14), and the effector functions of these cells can be modulated by type I interferons, IL-7, IL-15, and IL-23 (15). The review by Camard et al. discusses MAIT cells that are thymically pre-programmed to produce type 1 or 3 cytokines (i.e., MAIT1 or MAIT17 cells, respectively), the peripheral tissues they occupy, and their functions in tissue repair and cytocidal activity, which may be influenced by the specific cytokine milieu in which they reside, even under physiological conditions. The review then considers the contributions of these cells to bacterial and viral infections that are either dependent or independent of TCR engagement. Finally, they discuss the impact of human inborn errors of immunity, due to defects in genes related to MR1, RORγt and related cytokines and cytokine receptors, on MAIT cell functions and their contributions to health and disease.

Finally, the original research article by Zhang and Lu presents a bibliometric analysis of the iNKT cell literature in the Web of Science Core Collection (WSCC) database. Over three thousand studies were pared down by removing meeting abstracts, editorials, corrections, retracted papers, etc., to reach a final list of 2,579 studies that were analyzed for temporal distribution of publications, country of origin, institutional networks, journals, and specific scientists associated with top-ranked research. Such analysis can provide insight into the progression of a research field through specific milestones and collaborations, in efforts to make informed decisions about future research directions.

In summary, the articles contained in Volume III of this Community Series provide a snapshot of the biology and potential medical applications of CD1- and MR1-restricted T cells. We hope that these articles will spawn new studies with the ultimate goal to bring unconventional T cells from the lab to patient care.
